# A pulsed-air model of blue whale B call vocalizations

**DOI:** 10.1038/s41598-017-09423-7

**Published:** 2017-08-22

**Authors:** R. P. Dziak, J. H. Haxel, T.-K. Lau, S. Heimlich, J. Caplan-Auerbach, D. K. Mellinger, H. Matsumoto, B. Mate

**Affiliations:** 1 NOAA/Pacific Marine Environmental Laboratory, Newport, OR 97365 USA; 2 Cooperative Institute for Marine Science Studies, Oregon State University/NOAA, Hatfield Marine Science Center, Newport, OR 97365 USA; 3 Department of Geology, Western Washington University, Bellingham, WA 98225 USA; 4 Marine Mammal Institute, Oregon State University Hatfield Marine Science Center, Newport, OR 97365 USA

## Abstract

Blue whale sound production has been thought to occur by Helmholtz resonance via air flowing from the lungs into the upper respiratory spaces. This implies that the frequency of blue whale vocalizations might be directly proportional to the size of their sound-producing organs. Here we present a sound production mechanism where the fundamental and overtone frequencies of blue whale B calls can be well modeled using a series of short-duration (<1 s) wavelets. We propose that the likely source of these wavelets are pneumatic pulses caused by opening and closing of respiratory valves during air recirculation between the lungs and laryngeal sac. This vocal production model is similar to those proposed for humpback whales, where valve open/closure and vocal fold oscillation is passively driven by airflow between the lungs and upper respiratory spaces, and implies call frequencies could be actively changed by the animal to center fundamental tones at different frequency bands during the call series.

## Introduction

Over the last three decades, significant progress has been made in quantifying the signal characteristics and global variation of low-frequency sounds produced by blue whales, *Balaenoptera musculus spp* ^1–5^. Baleen whale vocalizations in general are low frequency (<1000 Hz)^1, 5–8^ and have been related to a variety of behaviors including feeding, navigation, mate attraction, and aggression^6, 9, 10^. As low frequency sounds travel much farther than high frequencies before attenuating, baleen whales likely use low frequencies to communicate over long distances^11–13^. Environmental factors such as an increased ambient noise from human activities may impact the range over which low frequency sounds can be detected^14^. Recent studies of blue whale calls suggest this has resulted in lowering the fundamental frequencies of calls over time, although several other factors have also been invoked such as increased blue whale population density or a gradual decrease in mean vocalization depth^3, 15^. The source of low frequency calls produced by a blue whale is currently thought to be resonance of the cavity spaces in the animal’s upper respiratory system^16–19^ which implies that body size is proportional to the frequencies generated (the larger the resonating organs, the lower the frequency of sound produced)^1, 2, 18, 19^. Therefore, understanding the internal physical processes behind sound production in blue whales, and how they regulate their call frequencies, provides important insights into how blue whale populations respond to environmental stressors such as increasing ocean noise. Since all blue whale populations are currently listed as endangered by the IUCN red list^20^, gauging blue whale response to changing marine environmental conditions is of utmost importance for their conservation.

## Blue whale call structure and resonance models

**Figure 1:**
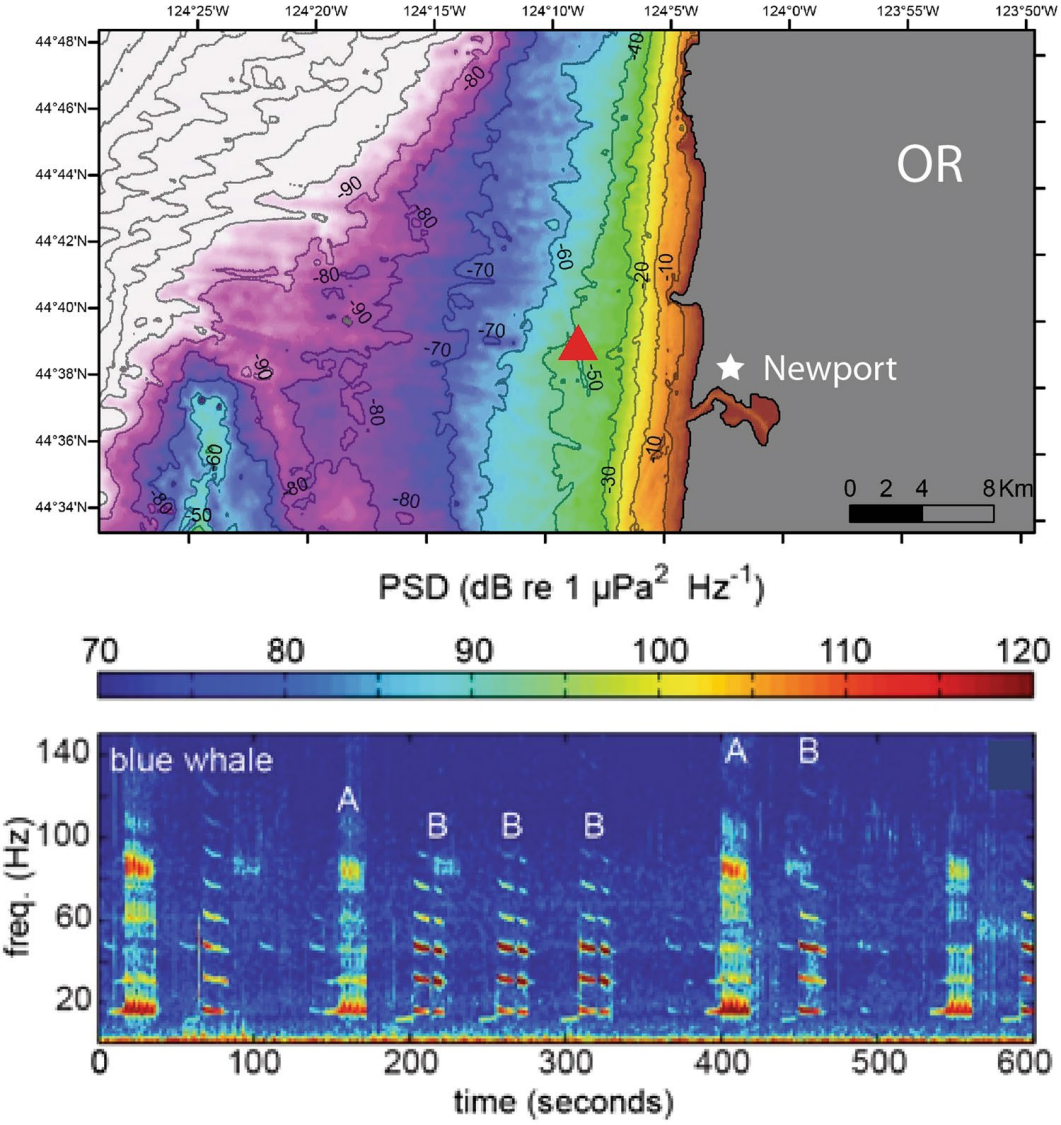
Top diagram shows location of hydrophone mooring (red triangle) ~8 km off the coast of Newport, Oregon. Seafloor depth contours are labeled. Bottom diagram shows example spectrogram of blue whale A and B calls recorded on the hydrophone (8 kHz sample rate) on 28 September at 01:57z (27 September at 18:57 PST). Bathymetric map was created using ArcGIS, [version 10.0], (http://www.esri.com/software/arcgis) software (Environmental Systems Research Institute) from a 100 m resolution xyz file of multibeam depth measurements, collected in 2011 by C. Goldfinger (Oregon State University, Active Tectonics Lab).

Blue whales have a distinctive call repertoire, with significant variability among populations. For example, northeastern Pacific blue whale calls (one of the best-studied studied populations) can be divided into four fundamental types, A, B, C, and D calls^18^. The A call, typically the first in a call series, consists of ~20 individual pulses less than 1 sec apart subdivided into multiple non-harmonic components (Fig. 1). The B call is a tonal harmonic signal typically 10–20 sec in duration with a fundamental frequency that steps down between 20 and 16 Hz (Fig. 1). The C call, is a 9–12 Hz upswept tone that precedes the B call. These three calls are often produced together in a pattern. The D call is a highly variable 80 to 30 Hz downswept signal 2–5 sec in duration that is often produced as a counter-call and not produced with the other call types^1, 4, 18^. The B call has traditionally been used to constrain sound production models because its long duration means the source mechanism is unlikely to involve re-circulated air to make the call^3^. B calls have reported source levels of up to 190 dB re 1 μPa based on recordings made off California^21^ most of the energy is in the first and third harmonics, where the energy distribution between the first and third is variable and either can be the most energetic in a given call.

Many questions exist with regards to the physics of baleen whale sound production mechanisms. Early work concentrated on bubble resonance, where a given volume of air (encased in an organ or chamber within the whale) at a given depth resonates at a fundamental frequency proportional to the chamber size^16, 17^. Aroyan *et al*.^18^ proposed that blue whales produce sound by a monopole source oscillation of an air-filled cavity, and the animal’s anatomy suggest that calls are due to Helmholtz resonance (i.e. resonance caused by airflow over an opening in a cavity or chamber), which allows for efficient constant frequency sound production over changing depths. There are many possible internal mechanisms for blue whales to radiate sound into seawater, where the oscillating source could be made of water, bone, or air. However, sound production systems that involve compressing water or dense tissue require much higher differential pressures and are therefore less efficient than air–filled systems^18^.

The primary energy source to cause airflow over a cavity or chamber would be the change in pressure produced as the animal rises and descends in the water column as it provides the volume displacements needed to produce the B call^3, 18^. It is generally assumed no air escapes from baleen whales during sound production^23^ because this would imply that water could enter the respiratory system and thus eliminate the whales’ ability to make the observed call series. The volume of air that can be held by adult blue whale lungs has been estimated at 2000 liters^3^. While a blue whale can provide a slight change in pressure through muscular compression of the lungs, it is unable to produce the 10 Bars of differential pressure at the sea surface needed to move this volume of air to the upper respiratory system^18^. Moreover, it seems likely that blue whales produce B calls only during ascent, eliminating the need to produce the positive differential pressure with its lungs that would be required during descent^18^. However, a study by Oleson *et al*.^24^ showed that the average change in depth during calling was <2 m, and therefore the animals are relatively stationary within the water column while vocalizing. Thus blue whales may not need to rise in the water column to reduce hydrostatic pressure and enable vocalizations. The lung volume assumed here would limit B call production depth to 90 m, which is consistent with field observations that indicate B calls are made at <30–40 m depth^18, 21, 24^, while maximum dive depths are estimated to be 300 m^25^. Air flow rates have been estimated at 25–50 l/s based on resonance quality factors and duration of B calls^18^.

## Anatomical sources of sound

**Figure 2:**
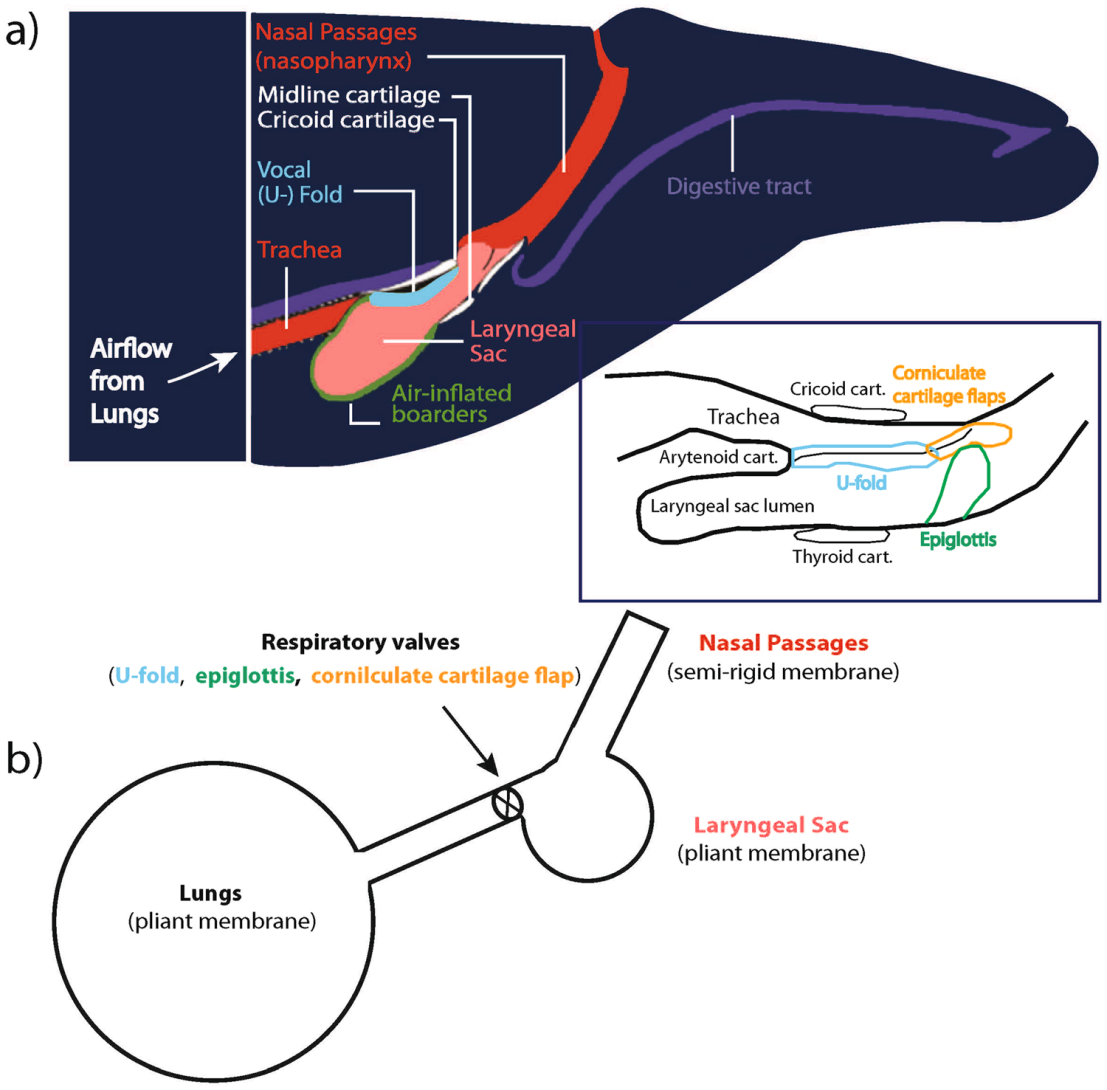
Schematic diagrams showing (**a**) generalized upper respiratory structures in a baleen whale (after *Reidenberg and Laitman* ^19^), with specific location and relative position of respiratory valves (after *Cazau et al*.^29^) in the attached insert. (reproduction permissions granted by John Wiley & Sons, Inc. and AIP Publishing LLC) and (**b**) simplified sound source model based on structures in (**a**) (after *Aroyan et al*.^18^; permission granted by Springer publishing), (**a**) Red area shows upper respiratory tract including nasal passages and trachea; purple shows the digestive tract; pink shows the laryngeal sac or lumen; green shows the air inflated borders of laryngeal sac; white shows midline, cricoid and thyroid cartilages; light blue shows the vocal or U-fold; the insert shows the position, relative to the U-fold, of the two other respiratory valves present in the laryngeal region (epiglottis and corniculate cartilage^29^) that also contribute to controlling airflow. Sound is thought to be produced by airflow from the lungs, through the oscillating, respiratory valves (the U-fold) then flow through the laryngeal sac and nasal passages. In the pulse model proposed here, repeated rapid adduction and dorsal elevation of the U-fold vocal process, against the cricoid cartilage, causes pulsed (restriction and release) airflow from the trachea to upper respiratory structures. Sound production likely originates by combining valve states and air recirculation by the lungs and the laryngeal sac through two opposite functions. The U-fold edges and laryngeal sac walls may also vibrate with airflow, potentially amplifying pulses. Laryngeal sac pulsed inflations may also cause pulsed distention of ventral throat grooves, facilitating sound transfer into water.

Previous studies have provided detailed anatomical information of the upper respiratory tract of baleen whales and used this as a basis to propose mechanisms for how baleen whales may produce their calls^26–31^. For example, in humpback whales, specific anatomical components and vocal production mechanisms have been related, and modeled, to show their relationship to particular acoustic call characteristics^29–31^. The source of vocalizations produced by baleen whales is thought to be via airflow within the respiratory system (Fig. 2a,b), where two symmetric lungs are connected to the trachea, a short, broad cone-shaped canal. In the laryngeal region, there are three respiratory valves: the epiglottis, the corniculate flaps and the U-fold. The U-fold possesses some strong similarities to common mammal vocal folds^19^, particularly in regards to its geometrical dimensions (length to thickness ratio) and slit-like glottal shape, tissue composition (presence of a thin mucosa in the outermost layer), and associated laryngeal muscles and cartilages (e.g., presence of homologous thyroid and arytenoid cartilages)^29^. Anterior to the U-fold (toward the nasal passages) is the epiglottis, whose valve function is to protect the lower respiratory system from foreign bodies (mainly water), and the corniculate cartilage flaps (Fig. 2a) characterized by its long shape, the high elasticity of its tissues, and the proximity of its two symmetric lips. The laryngeal sac can be seen as a soft extensible oval balloon with an extensive surrounding musculature. Opposite to the laryngeal sac, the nasal region is composed of the nasopharynx, a short flexible region of muscles and soft tissues, and two tube-like parallel nasal cavities with rigid walls which have thick plugs at the dorsal opening of the skull.

It is thought that the mechanism for low frequency sound production in baleen whales involves generating fundamental frequencies via U-fold vibration, followed by sound quality modification through variation of laryngeal parameters (by means of muscle contractions), and then sound transduction by means of laryngeal sac wall vibration to ventral throat pleat pulsations impinging on the surrounding water column^19, 29, 30^. Sound production likely originates by combining respiratory valve states (open when an airflow can pass through it, or closed otherwise) and air recirculation by the lungs and the laryngeal sac through two opposite functions (i.e. storing the incident airflow or emitting it back in the respiratory tractus)^29, 30^. This system would provide the air volume displacements needed to produce blue whale B calls whether or not the animal rises, descends, or remains relatively stationary within the water column. The strong muscular structures surrounding both the laryngeal sac and the lungs (i.e., respectively, abdominal muscles, the diaphragm, and the intercostal muscles) should pressurize the system to a specific pressure different from the depth related ambient pressure and allow it to withstand large variations in hydrostatic pressure while diving and surfacing. Thus airspaces inside the whale may be largely undisturbed by the changes in hydrostatic pressure with changes in the depth of the animal^29^.

The U-fold appears to be homologous to the vocal folds of terrestrial mammals based upon position, attachments, and composition^19^. Although oriented parallel to the airflow (versus the perpendicular vocal folds of terrestrial mammals), it nevertheless appears to have a similar function in airflow regulation and pneumatic sound generation. The U-fold structure is thick, lip-like, with numerous closely packed folds separated by narrow and often deep, irregular grooves, supported by arytenoid cartilages, and controlled by skeletal muscles^19^. This configuration of the folds is thought to lend great flexibility to the surface of the U-fold, permitting movements in different planes as well as the capability to stretch extensively and recoil back to the folded shape. When the lateral walls of the U-fold are approximated, they resemble two opposed lips. Airflow though the gap surrounded by the U-fold may cause the edges of these lips to vibrate, generating sound^19^. The U-fold’s orientation in the laryngeal lumen allows it to serve as a valve to narrow or seal the passageway for airflow between the upper and lower respiratory tracts. A thick, rigid U-fold may be expected, because thicker structures correlate with generating lower frequencies, and mysticetes vocalize in the low frequency range^19^. Thus, U-fold movements probably control sound output by varying U-fold thickness, length, tension, and the size of the gap between the U-fold’s lateral walls^19^. This movement of the U-fold structure has been invoked as another possible resonant source of sound in baleen whales^19, 29, 30^ where changes in vocal fold stretch/recoil (length, tension, and thickness) are necessary to achieve different frequencies. However, U-fold adduction/abduction and elevation/depression also control airflow and therefore the muscles that regulate the tension of the U-fold could control the production of short duration bursts of air, or pneumatic pulses, during airflow from the lungs to the upper respiratory passages or back from the laryngeal sac. A recent study modeling sound production sources of vocal nonlinearities in humpback whale songs^31^, show that fundamental frequency modulations are obtained by increasing (gradually) the stiffness factor of each side of the U-fold through modeling of the laryngeal muscle activity that controls the U-fold tension.

## Methods and Acoustic Vocalization Models

The fundamental frequency and overtones of a blue whale B call are thought to result from similar mechanisms proposed for humpback whales^29–32^, that is mainly from resonance due to flow of air through the U-fold, where flow induces self-oscillation of the U-fold leading to resonance whose spectral energy is then modulated by the nasal cavities and laryngeal sac. Early bio-acoustic signal-analysis research by Watkins^33^ showed that the variable repetition rate of sound pulses can be used to explain the harmonic frequency and spacing of dolphin clicks (e.g. *Tursiops truncatus*) and toadfish grunts (*Opsanus tau*). Moreover, it is known from seismo-acoustic source studies that a series of short duration pneumatic bursts, rather than Helmholtz resonance, can result in harmonic signals that vary in frequency. For example powerful, low-frequency harmonic acoustic signals can be produced by a rapid series of gas bubble bursts from a magma conduit in volcanoes^34, 35^ or frictional stick-slip movement of an iceberg keel plowing through the seafloor^36^. The mechanism behind these volcanic and cryogenic harmonic acoustic sources is the time spacing of the pneumatic or seismic pulse series, where a variation in the Δt spacing of the pulses results in a change in the fundamental and overtone frequencies, and/or the frequency spacing between the overtones.

**Figure 3:**
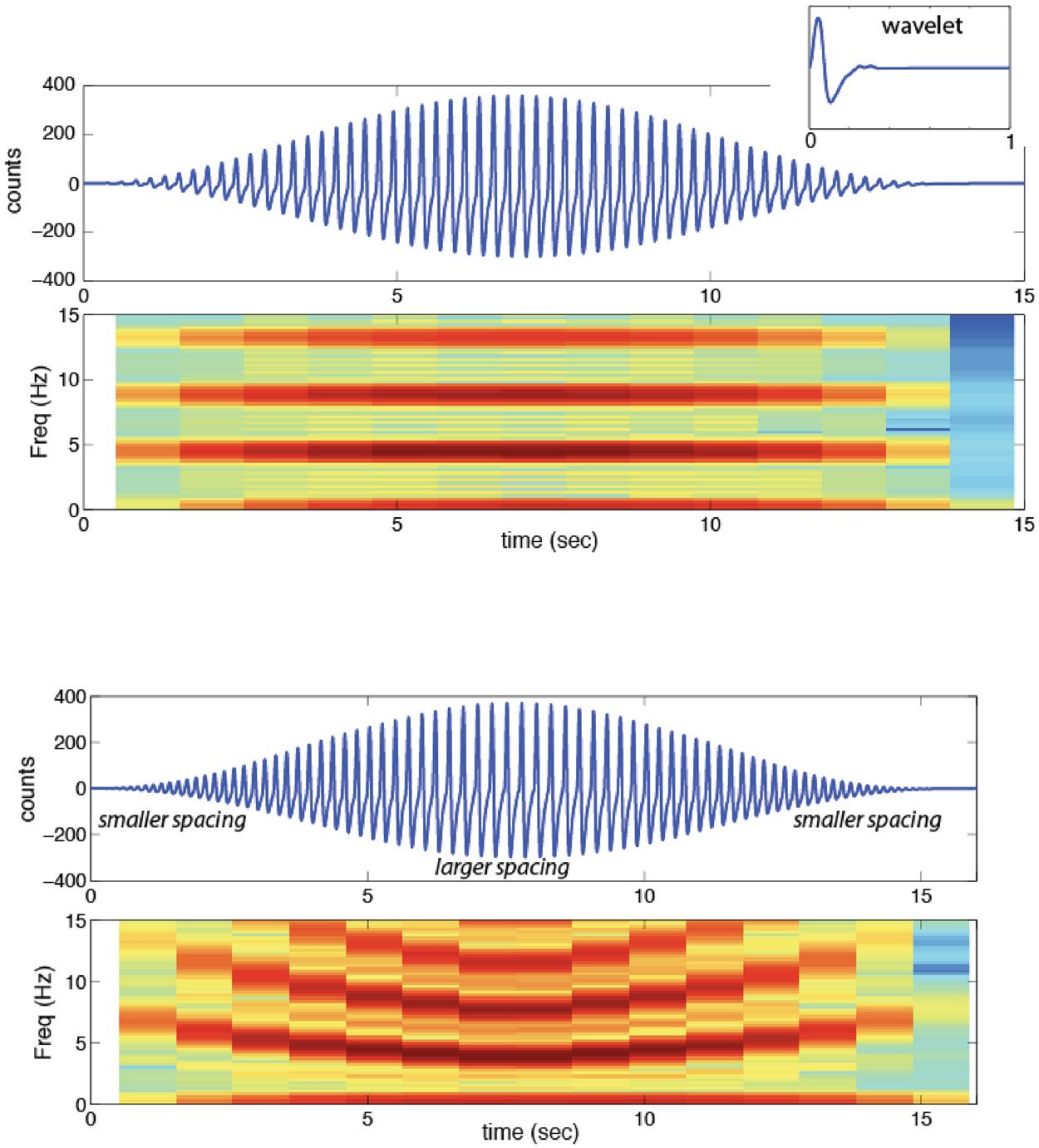
Example waveforms and spectra demonstrating wavelet model for generating repeating pulse harmonic signals. (**a**) Upper panel shows example of repeating pulse source model produced using a tapered amplitude sequence of wavelets of constant length. Upper right inset shows wavelet used (0.23 second for this demonstration). A series of these wavelets are aligned using constant inter-event time to construct the waveform below. A cosine taper is also used on the pulse series waveform. The frequency and overtones in the spectrogram of the waveform are inversely proportional to the time spacing of the maximum amplitude of the wavelets. (**b**) Upper panel shows a tapered amplitude sequence of wavelets with variable inter-event time (range from 0.16–0.23 sec). Lower panel shows resulting spectrogram with shifting frequencies due to change in wavelet time length in original signal.

An example of how repeating wavelets can produce harmonic signals is shown in Fig. 3a, where a short duration pulse is repeated in series to generate a longer duration signal. A series of these wavelets (shown in inset) were then aligned using a constant inter-pulse time to construct the 15 sec long waveform, with a cosine window used to taper the signal. The fundamental frequency and overtones observed in the resulting spectrogram of the pulse-series waveform are inversely proportional to the time spacing of the maximum amplitude of the wavelet series. By varying the inter-pulse interval, Fig. 3b shows that the fundamental and overtone frequencies of the waveform are changed, while the harmonic frequency spacing remains the same.

**Figure 4:**
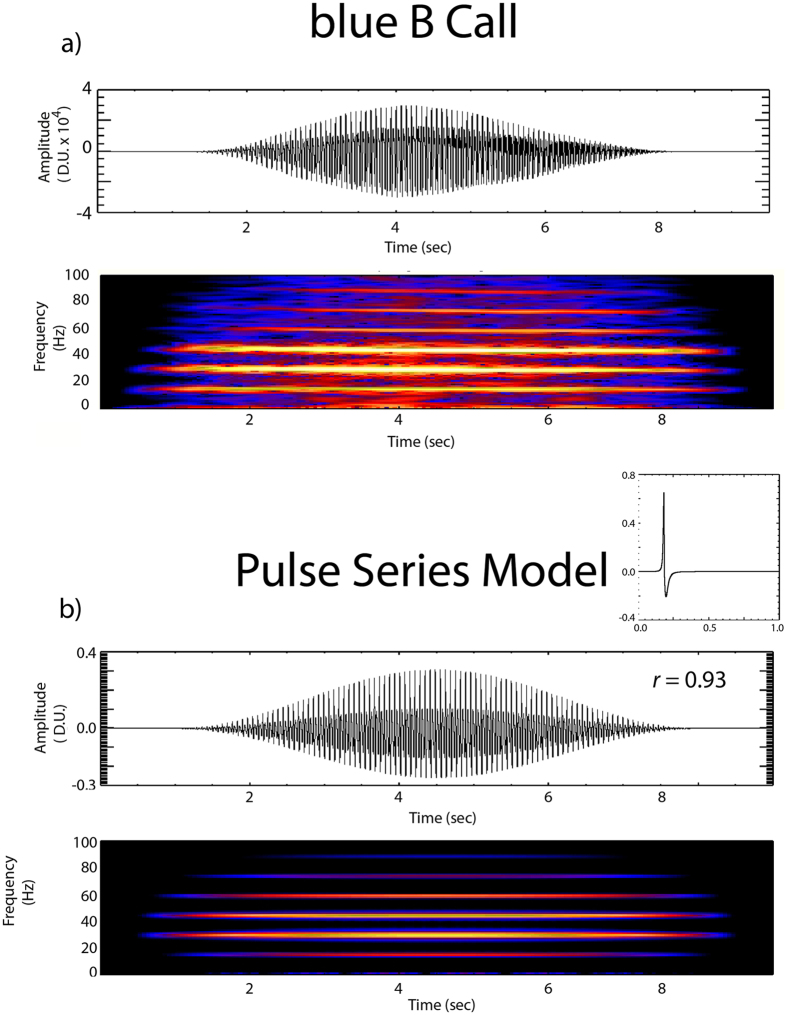
(**a**) Top panel shows small scale plot (7 sec duration) and corresponding frequency spectra of blue whale B call from Fig. 1. Note fundamental at 14.73 Hz with multiple overtones. (**b**) Top panel shows repeating pulse model with source wavelet (0.18 sec duration) used to produce time series on upper right. This repeating pulse model of a blue whale B call was produced by 8000 successive wavelets and tapering the amplitude sequence. Pulse model was cross-correlated with original B call with resulting *r* shown.

A detailed display of the blue whale B call from Fig. 1 is shown in Fig. 4a. This B call was recorded using a sample rate of 2 kHz, and has a fundamental frequency of 14.7 Hz with 5 overtones at 29.5, 44.2, 58.9, 73.6, and 88.4 Hz.

To model this B call, we first constructed a short duration pulse *P(t)* using the equation:

1 $$P(t)=\frac{dq}{dt}$$

where,

$$q(t)=1/[{(t-a)}^{2}+0.01)];\,{\rm{for}}\,{\rm{t}} < a$$

and

$$q(t)=10/[{(t-a)}^{2}+0.1)];\,{\rm{for}}\,{\rm{t}} > a$$

where *a* is a constant value used to set the time length of the pulse. Here we set *a* = 0.3 so that t > 0 and the pulse duration remains under a second. The upper right of Fig. 4b shows the 0.068 sec duration pulse wavelet produced using equation (1). This pulse was then repeated in series 3081 times to construct the 7 sec long B call waveform model shown in Fig. 4b.

### Model comparison and acoustic propagation

**Figure 5:**
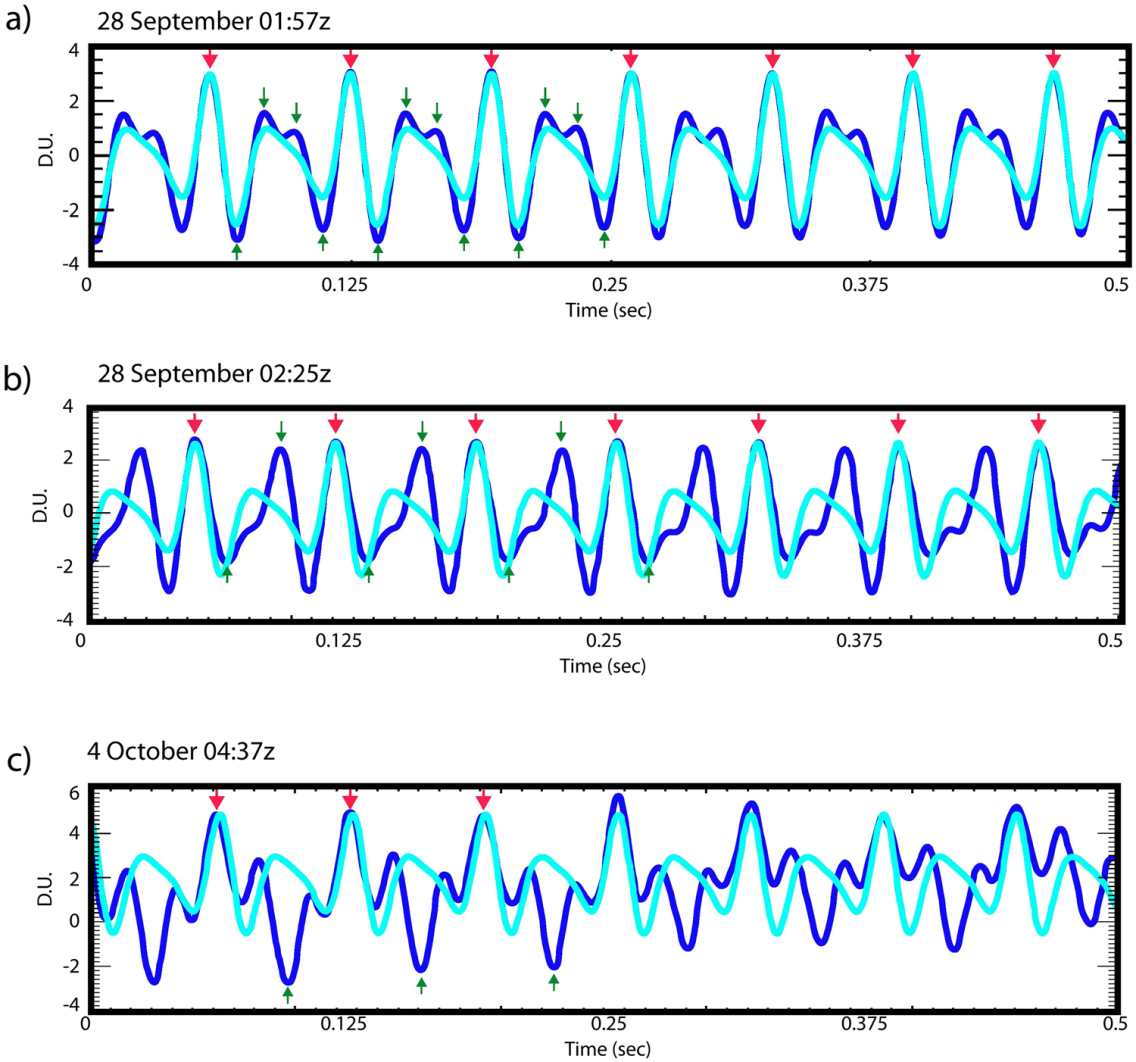
Three detailed records of B calls recorded by a hydrophone (dark blue) and model of B call produced using eq. 1 (light blue). Signal (**a**) is from 28 September (same signal as in Fig. 1), (**b**) is ~30 min after (**a**), and (**c**) is call recorded 6 days later. Arrows point at significant components of the actual B call signal (dark blue). Red arrows show the fundamental frequency of the B call which is the direct arrival phase (the acoustic phase with a direct path from the whale to the hydrophone). Green arrows highlight later arriving, lower amplitude signal components that include the call overtones. Time delays as well as amplitude and polarity changes are introduced into these lower amplitude signal components by propagation effects such as reflection off nearby seafloor structures (e.g. reefs, sand bars) or possibly cavities or bony structures within the whale. Signal (**a**) shows best match of model to real blue call. Signals (**b**) and (**c**) show more mismatch in signal width, amplitude and polarity of later arriving phases possibly due to changing position and orientation of the whale relative to seafloor structures. Ocean noise levels are also much higher before and after 28 September which likely contributes to mismatch of model in (**b**) and (**c**).

To test the quality of the model fit, we cross-correlated a blue whale B call recorded on a seafloor hydrophone at 55 m depth (Fig. 4a) with the model waveform (Fig. 4b). A more detailed comparison of the observed and model call signals is also shown in Fig. 5a. The example B call presented here is a very clear signal with little high frequency noise interference. Both Figs 4 and 5 show remarkable similarities in amplitude, phase difference, pulse width and polarity between the blue whale call and the pulse model. The repeated, high amplitude phase seen in the call and in the model is the fundamental frequency of 14.73 Hz (0.068 sec time spacing, labeled with black arrows). Moreover, the resulting correlation function *r* of 0.93 (where a maximum correlation = 1) indicates our pulse model well matches the B call signal structure.

Admittedly, by adjusting the time length of the pulse (the value of *a* used above), we are employing a forward modelling methodology (subjective) where source metrics are chosen that allow us to best match the model to the real signal, as opposed to discrete inverse methods that use the recorded signal to objectively derive the source parameters. We employed a forward modelling approach in this study because, while there are several physiological aspects to sound production in humpback whales that have been previously determined and modeled^19, 29–31^, the precise physical mechanism or process that produces the amplitude and pulse width of the observed blue whale B call waveforms is not fully known. Thus a forward model is our attempt to provide a new approach and different view of the B call source mechanism. The time duration of air pulses that produce blue whale B calls may be controlled by a wide variety of physiological factors that could vary between individual animals (e.g. respiratory valve thickness, tissue elasticity, strength of controlling muscles, etc). Adjusting the pulse time-decay parameter *a* in the model to match the observed signal is a way to account for this wide range of variables in the actual sound generation mechanism.

Closer inspection of Fig. 5a also shows the call has lower amplitude components with pulse widths and polarities that arrive between the peaks of the 14.73-Hz fundamental (highlighted with green arrows). These time-delayed, lower amplitude arrivals are also observed in B calls recorded at different times within the acoustic data set. Figure 5b and c show examples of other B calls recorded ~30 min later than the call in Fig. 5a and a call recorded 6 days later.

These lower-amplitude signal components in between the fundamentals have arrival times that do not match the expected time delay for discrete overtones. For example, the lower amplitude phases in Fig. 5a have time delays of 0.03 and 0.04 secs after the fundamental, representing 37.6 and 24.5 Hz, respectively, whereas expected overtones should be at integer multiples of the fundamental frequency; 29.5 Hz, 44.2 Hz, etc. Therefore, while these low amplitude arrivals do include the signal energy from the B call overtones, the time delays and lower amplitudes are likely caused by multipath effects from the signals reflecting/refracting off the sea-surface, seafloor and/or reef structures in this shallow water propagation environment. Moreover, because the model matches the observed B call so closely in Fig. 5a suggests the multipath effects from this particular instance are not strong, which may mean the whale was relatively close to the hydrophone at the time of this recording. In Fig. 5b,c, our model does not match the recorded B call as well, suggesting the whale was farther from the hydrophone receiver resulting in more complex propagation. Producing model estimates of the propagation paths for rays travelling from the blue whale to the hydrophone in order to map the arrival times of the various components of the B call signal structure is challenging in this shallow water environment. Since the depth of the hydrophone (~55 m) is much less than the wavelength of the B call (~100 m), a ray approximation model that yields arrival time estimates (e.g. Bellhop^37^), will not produce valid estimates of the multipath interference^38^.

However, it still seems highly likely that this mismatch in the non-fundamental components of the actual B call and the model may be due to time delays and amplitude/polarity changes due to reflections from the seafloor or nearby structures (e.g. reefs, sand bars). Evidence that these phases may be reflections comes from the observation that these components of the actual blue whale call have very different signal characteristics when recorded on different days. For example, as noted, Fig. 5a shows the best match of both fundamental and overtones with our model to an actual blue whale B call, whereas Fig. 5b and c show the same strong correlation in the fundamental frequency of the data and model, but now accompanied by a much greater degree of mismatch in signal width, amplitude and polarity for the overtones of these later arrivals. These mismatches between actual data and model might be due to variability of the real signal caused by the arrival of acoustic reflections off nearby seafloor structures as the blue whale approached, or even as the animal changed orientation or position in the water column relative to these structures. Moreover, ambient noise levels in the ocean, recorded on the hydrophones, were much higher before and after 28 September due to increased wind speeds and sea-surface wave heights. Ambient ocean noise generated by wind and surface waves is known to be broadband centered at 200 Hz^39^ but can go as low as <10 Hz^40^, even in shallow water. We think it is also possible that the discrepancies between the model and observed calls in Fig. 5b,c may be due to relatively high background noise levels, which will be present in the observed signal despite filtering because the noise and the whale call occur in the same frequency band

The non-fundamental signal components may also result, in part, because the blue whale is not a “perfect” signal generator. The waveforms produced by any natural system will have internal reflections from anatomical structures and/or cavities within the animal itself. These ancillary sound sources from within the animal can be further complicated by acoustic propagation effects within the water-column. Although, as noted by Reidenberg and Laitman^19^, once the sound is generated inside the animal the signal is likely not distorted much by propagation through the animal’s tissues and throat pleats, since the density of the tissues and pleats is closer to the density of sea water than air.

If high-resolution bathymetry were to be collected in the vicinity of the hydrophone, it might be possible to identify seafloor structures that act as these acoustic reflectors. However, without the exact three-dimensional path of the blue whale through the water-column, it is not possible to precisely calculate the propagation path and arrival time of these reflections in order to identify these phases in the B call signals.

### Comparison to Resonance Based Source Model

**Figure 6:**
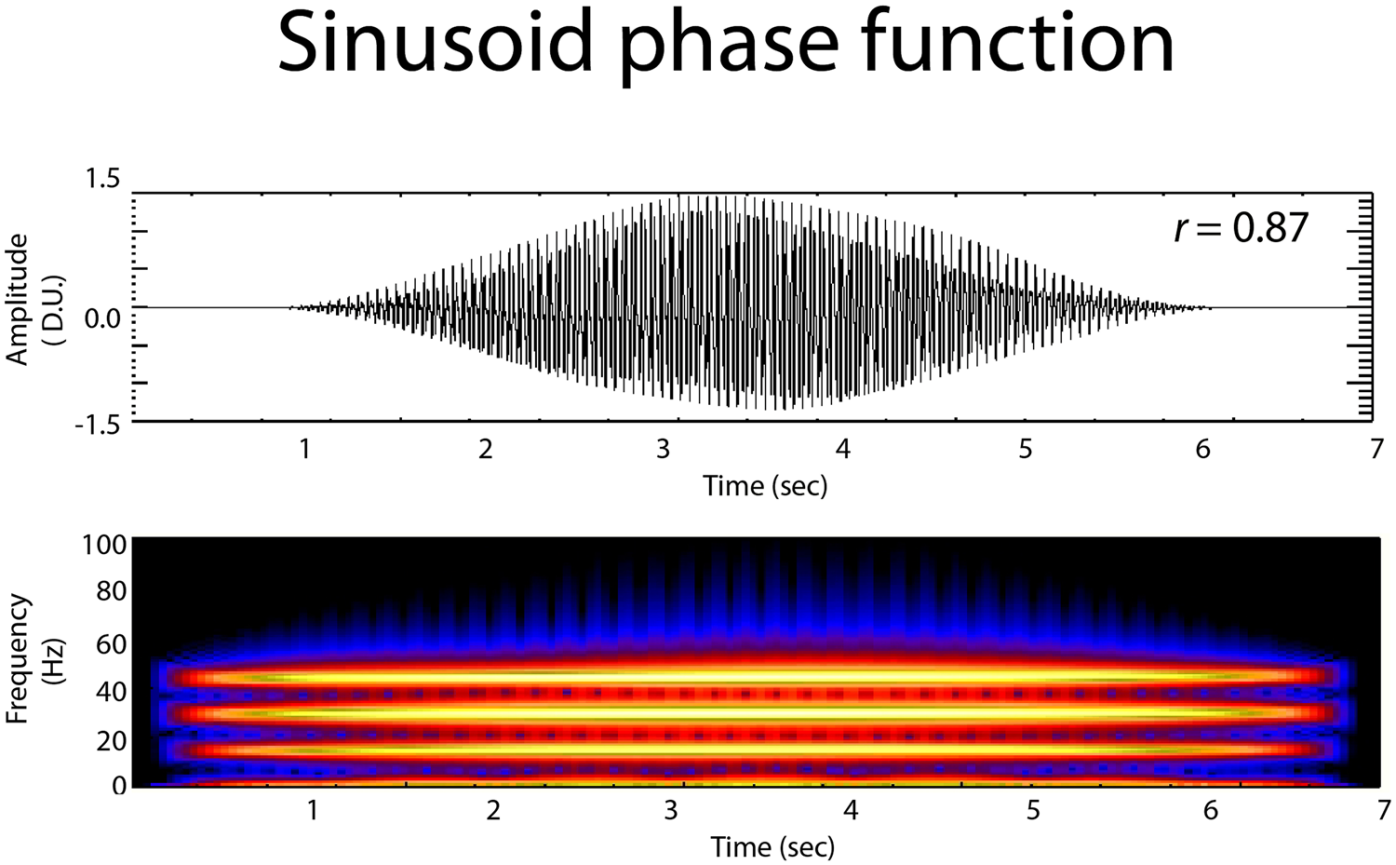
Example of a sinusoidal based blue whale B call model. The model here is a sine function summation, but with a variable phase term introduced (eq. 3) to better improve the match of the width of individual waveforms^38^. The model was cross-correlated with an original B call, and resulting *r* is shown.

Previous models of blue whale calls have been based on either summation of sinusoids^41, 42^ or Gaussian curves^43^. A key distinction between our pulse model and previous sinusoidal/Gaussian models is that the latter is characteristic of a resonance source mechanism. To compare the match between our pulse and resonance-based models, a sinusoidal based blue B call model is shown in Fig. 6. The model developed by Mellinger *et al*.^41^, is a sine function summation but with a variable phase term introduced to better improve the match of the width of the model individual waveforms. This model dynamically changes the amplitude and frequency in the waveform, and is the basis for animal and non-animal signal detection algorithm that tracks the spectral peaks of the call frequencies over time. The algorithm function SP is given by:

2 $${\rm{SP}}={\sum }_{(i=0)}^{(N-2)}[{{\rm{A}}}_{0}+i({{\rm{A}}}_{1}-{{\rm{A}}}_{0}){({\rm{N}}-1)}^{-1}]\sin ({\rm{P}}+2\pi {{\rm{R}}}^{-1}\,[{\sum }_{j=0}^{i}j({{\rm{F}}}_{1}-{{\rm{F}}}_{0})/{\rm{N}}+{{\rm{F}}}_{0}])$$

where R is the sample rate, P is the phase (here set to 0), and N is the number of data points (product of R and the duration in seconds). Also F_0_ and F_1_ are the observed fundamental and overtones frequencies (in this case 14.73, 29.5 Hz) but varied at ±0.42 Hz, respectively, to simulate the slight frequency sweep of the tones. The values for A_0_ and A_1_ are the amplitude coefficients used to keep the signal range between ±1.5 and to adjust for variation in signal amplitude.

As with the pulse model (eq. 1), this sine-based algorithm (eq. 2) was cross-correlated to the actual B call signal to gauge the accuracy of the model fit. Correlation of the function in eq. 2 with the B call in Fig. 6 results in a *r* value of 0.87, indicating a good match of this model to the actual call. However, the pulse series model (eq. 1) still appears to do the best job of simulating the B call, as indicated by the higher correlation *r* value of 0.93, which lends support to our hypothesis that a series of air pulses may likely involved in the formation of a blue whale B call. Nevertheless, the high correlation of the sinusoid phase delay model (eq. 2) indicates that internal resonance within the animal may also contribute to generation of the B call.

## Discussion

We suggest that blue whales produce B calls by rapid opening and closing of one of their respiratory valves, which yields a series of pneumatic air bursts or pulses. This opening and closing of respiratory valves is likely passively triggered by the physical interaction of airflow (through lungs and upper respiratory structures) and U-fold tissues, i.e. the self-oscillating flow-induced vocal production model proposed by Cazau *et al*.^29, 31^ for humpback whales. These pneumatic bursts, taken in series, produce the fundamental and overtone frequencies of the B call. This signal process is demonstrated by the models shown in Figs 3 and 4, where the frequency and overtones of the B call are inversely proportional to the time spacing of the maximum amplitude of the pneumatic pulses. Moreover, any variation in this inter-pulse time spacing will result in a shift of the fundamental and overtone frequencies through time that also maintains the frequency spacing between the overtones. Thus similar to previous models for humpbacks^29, 31^, the pulse model described here also allows for changes in frequency of the B call through time as observed in actual calls.

We further suggest that these pneumatic air pulses can be produced, as outlined in Cazau *et al*.^29^ for humpback whales, by one of three physiological configurations for the laryngeal respiratory valves. In our view, the main source of the pneumatic pulses is likely from repeated rapid adduction and dorsal elevation of the U-fold vocal process against the cricoid cartilage, causing pulsed (restriction and release) airflow from the trachea into the laryngeal sac and upper respiratory structures^19, 30^. This adduction is likely passively driven by airflow as is thought to be the case for humpbacks^29^. However, the corniculate cartilage and the epiglottis are also respiratory valves and will open and close in association, and in various configurations, with the U-fold^29^. Thus all three valves may take turns being either the main valve controlling the rate and duration of the pneumatic air bursts, or may coordinate with the other vales in controlling the pulse of air. In addition, the U-fold edges and laryngeal sac walls may also vibrate and resonate with airflow, potentially amplifying these pulse signals^19^. Frequency modulation of the air pulses may also result from the inflation of the laryngeal sac, stretching in correspondence the U-fold through the particular free-floating attachment between its lumen and the arytenoid muscle, instead of being produced through the activity of dedicated laryngeal muscles^19, 29^. Pulsed inflation of the laryngeal sac may also cause pulsed distention of ventral throat grooves, facilitating sound transfer into the water^19^. While our pulse model implies that harmonic sounds of blue whales are primarily produced by pneumatic airflow through the respiratory valves, the laryngeal sac and nasal passages likely ﻿still﻿ produce resonance sound during airflow, adding to the overall B call energy and structure, and/or contributing to the generation of observed secondary pulses.

The pulse series model also implies that the whale can change the frequency of the B call while the call is being created, which may account for several examples of unusual B calls previously observed. For example, Stafford and Moore^44^ showed the existence of an atypical B call series recorded from blue whales in the north Pacific. The unusual B call pattern consisted of an abrupt rise in frequency within the call. Stafford and Moore^44^ attributed this unusual record to either an individual producing a hybrid song that included both northeastern and northwestern blue whale call units and/or two individuals from either population vocalizing in perfect synchrony with each other. Although we cannot rule out two individuals vocalizing, the unusually rapid change in call frequency could be due to a blue whale cognitively changing the time between pneumatic air pulses and, therefore, changing the frequency within the call. Similarly, Thode *et al*.^22^ showed examples of Channel Island blue whale B calls that had unusual U-shaped frequency modulation in addition to the well-established down-sweep. Thode *et al*.^22^ attributed these call structures to mechanical resonance within the animal, but noted the vocalizing-depth to frequency-shift relationship does not fit any simple air-filled Helmholtz resonator model.

Beginning in the early 1990s, a unique series of whale calls were observed centered at 52 Hz, ~6 sec in duration, with no under- or overtones^44, 45^. Watkins *et al*.^45^ attributed this to an unknown large whale species, while Stafford and Moore^41^ observed that the call shared characteristics with the third harmonic of eastern north Pacific blue whale B calls and therefore may be part blue whale or perhaps a blue-fin hybrid. It is not uncommon to observe northeastern Pacific blue whale call series where the fundamental and first overtone of the B call are not recorded and only the third harmonic of the call is observed^44^. A simple resonating source model cannot account for removal of fundamental tone energy, and still allow high energy production of the high overtone. The pulse model presented here provides a physical mechanism, as does the Cazau *et al*.^29, 31^ model for vocal production in humpback whales, whereby call frequencies could be actively changed by the animal to center fundamental tones at different frequency bands during the call series.

McDonald and Hildebrand^3^ observed that northeastern Pacific blue whale songs, measured at the fundamental frequency and third harmonic, have been exhibiting a long-term linear downward trend and are now 31% lower in frequency than they were in the 1960s. They suggest this downward shift is related to increased population size post commercial whaling, modified by trade-offs between short- and long-distance communication and global ambient noise increases due to shipping. A similar long-term decrease in vocalization frequencies of both Antarctic blue and eastern Indian Ocean pygmy blue whales has also been observed^15, 46^. We speculate that our pulse model may provide a viable mechanism to account for this observed change in frequency whereby blue whales are able to cognitively lower their call frequency to adjust for long term changes in the ocean soundscape and their environment.

Despite the difference in physical mechanisms between a purely resonance and pulse model of sound production in blue whales, the previously proposed limits on a whale’s B call structure and pattern imposed by maximum dive depth, inhalation, and feeding would still apply^18^. Global observations of tonal blue whale calls^24, 47, 48^ show sound level intensity of these calls is relatively constant over the duration of the call, indicating there is a near constant air flow rate from the lungs across the respiratory valves during the call production. Moreover, it might take less energy for the whale to vocalize during dive or ascent because the change in hydrostatic pressure may facilitate the flow of air from and/or into the lungs across the respiratory valves, allowing the animal to maintain the sound intensity over the typical time duration of B calls (~10–20 sec). Assumptions of lung and laryngeal sac volumes imply sound production at a maximum dive depth limit of <200 m^3, 18^, although models of airflow rates show vocalization depths may range from 0–90 m, consistent with observed vocalization depths of 10–40 m^22^. Thus these depth limits and differential pressure restrictions are still applicable to our proposed pulse model because the model is based on the whale’s ability to maintain a high airflow rate across the oscillating, respiratory valves.

While the pulse model presented here is still in the preliminary stages of development, our first-order analysis indicates the model may provide an alternative mechanism to explain unusual blue whale call characteristics that cannot be fully explained by resonance of air-filled organs within the animal. The pulse model may also be applied to vocalizations of other baleen whale species or marine animals to help understand how oddly-low frequency vocalizations can be produced by animals of small body size. For example, even though Atlantic cod (*Gadus morhua*) are typically <1 m in body length, they produce low frequency harmonic spawning sounds in the 50–150 Hz range^49^. These frequencies require sound wavelengths, as well as a resonating source dimension, on the order of 10–30 m. Hence an internal pneumatic pulsed air mechanism may help explain the low frequency vocalization characteristics of these animals.
